# Development of nerves and vessels in the penis during the human fetal period

**DOI:** 10.1590/S1677-5538.IBJU.2024.9916

**Published:** 2024-08-10

**Authors:** Carla B. M. Gallo, Waldemar S. Costa, Luciano A. Favorito, Francisco J. B. Sampaio

**Affiliations:** 1 Universidade do Estado do Rio de Janeiro Unidade de Pesquisa Urogenital Rio de Janeiro RJ Brasil Unidade de Pesquisa Urogenital, Universidade do Estado do Rio de Janeiro - UERJ, Rio de Janeiro, RJ, Brasil

**Keywords:** Blood Vessels, Penis, Fetus, Humans

## Abstract

**Introduction:**

Although nerves and vessels of the penis play important role in erection, there are few studies on their development in human fetus. Therefore, the objective of the present study is to analyze, quantitatively, in the corpora cavernosa and corpus spongiosum, the development of the nerves and vessels in the fetal penis at different gestational ages.

**Material and Methods:**

Fifty-six fresh, macroscopically normal human fetuses aged from 13 to 36 weeks post-conception (WPC) were used. Gestational age was determined by the foot length criterion. Penises were immediately fixed in 10% formalin, and routinely processed for paraffin embedding, after which tissue sections from the mid-shaft were obtained. We used immunohistochemical staining to analyze the nerves and vessels in the corpus cavernous and in the corpus spongiosum. These elements were identified and quantified as percentage by using the Image-J software.

**Results:**

The quantitative analysis showed that the percentage of nerves varied from 3.03% to 20.35% in the corpora cavernosa and from 1.89% to 23.88% in the corpus spongiosum. The linear regression analysis indicated that nerves growth (incidence) in the corpora cavernosa and corpus spongiosum correlated significantly and positively with fetal age (r2=0.9421, p<0.0001) and (r2=0.9312, p<0.0001), respectively, during the whole fetal period studied. Also, the quantitative analysis showed that the percentage of vessels varies from 2.96% to 12.86% in the corpora cavernosa and from 3.62% to 14.85% in the corpus spongiosum. The linear regression analysis indicated that vessels growth (appearance) in the corpora cavernosa and corpus spongiosum correlated significantly and positively with fetal age (r^2^=0.8722, p<0.0001) and (r^2^=0.8218, p<0.0001), respectively, during the whole fetal period studied. In addition, the linear regression analysis demonstrated a more intense growth rate of nerves in the corpus spongiosum during the 2nd trimester of gestation, when compared with nerves in the corpora cavernosa. In addition, the linear regression analysis demonstrated a more intense growth rate of vessels in the corpus spongiosum when compared with the corpora cavernosa, during the whole fetal period studied.

**Conclusions:**

In the fetal period, the human penis undergoes major developmental changes, notably in the content and distribution of nerves and vessels. We found strong correlation between nerves and vessels growth (amount) with fetal age, both in the corpora cavernosa and corpus spongiosum. There is significant greater proportional number of nerves than vessels during the whole fetal period studied. Also, nerves and vessels grow in a more intense rate than that of the corpora cavernosa and corpus spongiosum areas.

## INTRODUCTION

The urinary and genital systems have the same embryological origin and are derived from the intermediate mesoderm. The general plan of vertebrate development is very similar and well known since the 19th century, however, little is known about human fetal development, especially in relation to the penis and its components.

The erectile tissue of the human penis is composed of elastic fibers, collagen fibers, smooth muscles, arteries and veins, and has important functions in the mechanism of penile erection (1–5).

Some works from our group have shown the characterization of morphological components of the penis during embryonic and fetal development (6–11). Histochemical and immunohistochemical analyses, of which some were associated with morphometry, have characterized structural components in the erectile tissue of adult penis (3, 4, 12), and, in preliminary works, these techniques have been used to investigate erectile tissue in the human fetal penis (4, 6). The knowledge of such structures is necessary for understanding the normal physiology of the adult penis, commonly altered in different clinical or experimental situations (12, 13, 15). Therefore, it is important to know the changes of these penile structures during the human fetal development.

Recently, it has been demonstrated the development of the penile, the corpora cavernosa and the corpus spongiosum areas, during the human fetal period (10). Also recently, it has been studied the morphology, development, modifications and distribution of the erectile tissue in the fetal penis (11). Nevertheless, despite nerves and vessels present an essential role in erection, there are few or even no studies on its development in the penis of human fetuses.

Therefore, the objective of the present work is to analyze, qualitative and quantitatively, in the corpora cavernosa and corpus spongiosum, the development of nerves and blood vessels during the whole fetal period (13 to 36 weeks post-conception – WPC), providing normative patterns of growth.

## MATERIAL AND METHODS

The study protocol was approved by the ethical committee on human research at our institution.

Our analysis was done every 15 days, during the 2nd and 3rd trimesters of pregnancy. The analysis began from the 13th week, when the characteristics of the main elements of the corpora cavernosa and corpus spongiosum were already present.

We studied 56 penises from fresh normal human fetuses. All fetuses had died of causes unrelated to the urogenital tract. The fetuses were well preserved, and none had any detectable congenital malformation. Gestational age ranged from 13 to 36 weeks post-conception (corresponding to 15 to 38 menstrual weeks) and was estimated by the foot length criterion (16–19). The fetuses were dissected with a magnification glass, and the urogenital bloc containing kidneys, ureters, bladder, prostate, testes and penis was removed. We used 1 to 5 fetuses of each gestational age.

After dissection, the penis was incised at the pubic symphysis, around 2 mm from it, cross-sectioned at its mid shaft and fixed in 10% formalin, prepared in PBS for 24 hours and routinely processed for paraffin embedding and sectioned at 5-μm with intervals of 200-μm between each section.

We used immunohistochemistry methods to analyze the nerves and vessels in the corpora cavernosa and in the corpus spongiosum. Endothelial cells were detected by using a primary antibody anti-CD31 (Abcam, Cambridge, MA, USA) at a dilution of 1:30. An anti-tubulin (Zymed Lab, Carlsbad, California) with Histostain-Plus Kit secondary antibody (Invitrogen Immunodetection, Camarillo, California) was used for characterization and quantification of nerves. Histological images were captured on a digital camera (DP71, Olympus, Tokyo, Japan) coupled to a light microscope (BX51, Olympus). These elements were identified and quantified as percentage by using the Image-J software.

Statistical Analysis - With the aid of GraphPad Prism® 5.0 software, by using the mean values for each fetus, we performed the statistical analysis by simple linear regression, assessing the association between the variables analyzed with fetal age and other variables. Also, the correlation coefficient (r^2^) and p-value were obtained for each regression analysis, with p ≤ 0.05 considered significant.

## RESULTS

**Figure 1:**
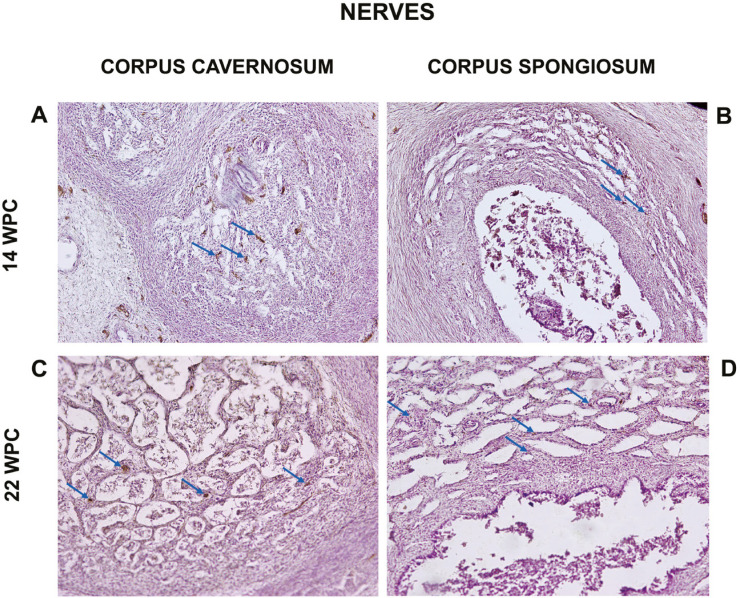
Photomicrographs showing: A and C: Nerves in the corpora cavernosa (arrows). A) Fetus with 14 weeks post-conception (WPC) and C) Fetus with 22 WPC. B and D: Nerves in the corpus spongiosum (arrows). B) Fetus with 14 WPC and D) fetus with 22 WPC. Immunohistochemistry for anti-tubulin-β3, X200.

**Figure 2:**
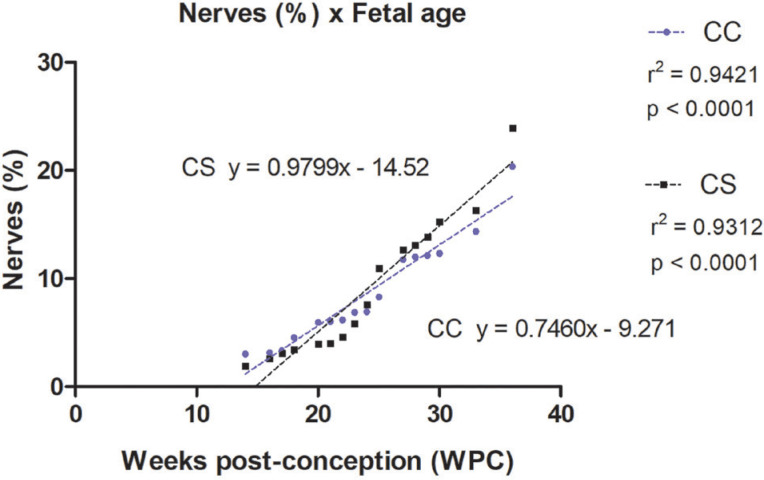
Linear regression analysis showing the percentage of nerves in the corpora cavernosa (CC) and corpus spongiosum (CS), according to the fetal age in weeks post-conception (WPC).

The quantitative analysis showed that the percentage of nerves varied from 3.03% to 20.35% in the corpora cavernosa and from 1.89% to 23.88% in the corpus spongiosum. The linear regression analysis indicated that nerves growth (incidence) in the corpora cavernosa and corpus spongiosum correlated significantly and positively with fetal age (r^2^=0.9421, p<0.0001) and (r^2^=0.9312, p<0.0001), respectively, during the whole fetal period studied (Figures 1 and 2).

**Figure 3:**
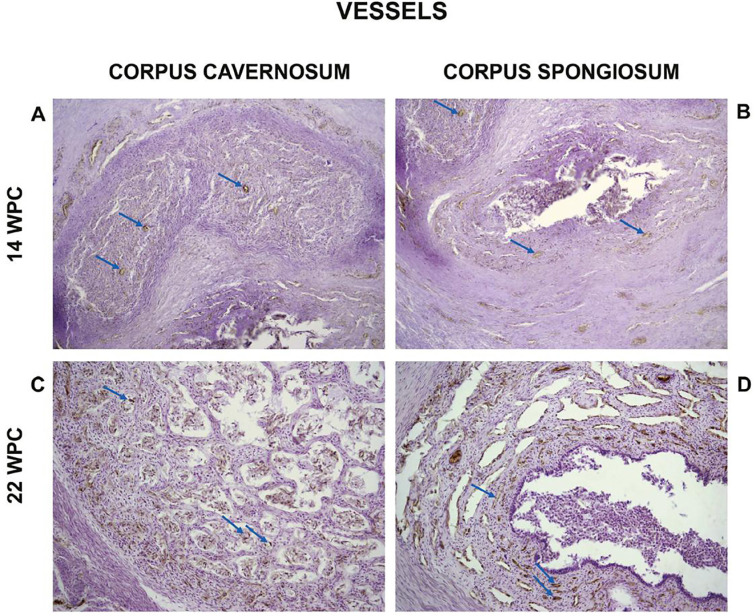
Photomicrographs showing: A and C: Vessels in the corpora cavernosa (arrows). A) Fetus with 14 weeks post-conception (WPC) and C) Fetus with 22 WPC. B and D: Vessels in the corpus spongiosum (arrows). B) Fetus with 14 WPC and D) fetus with 22 WPC. Immunohistochemical for anti-alpha-actin of smooth muscle, X200.

**Figure 4:**
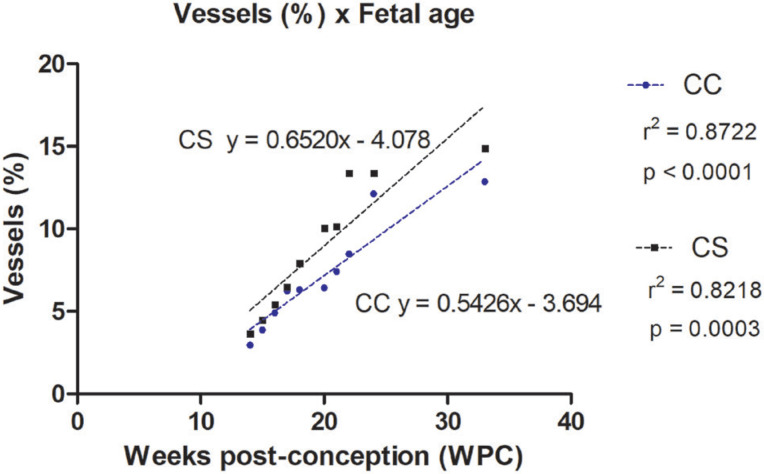
Linear regression analysis showing the percentage of vessels in in the corpora cavernosa (CC) and corpus spongiosum (CS), according to fetal age in weeks post-conception (WPC).

Also, the quantitative analysis showed that the percentage of vessels varies from 2.96% to 12.86% in the corpora cavernosa and from 3.62% to 14.85% in the corpus spongiosum. The linear regression analysis indicated that vessels growth (incidence) in the corpora cavernosa and corpus spongiosum correlated significantly and positively with fetal age (r^2^=0.8722, p<0.0001) and (r^2^=0.8218, p<0.0003), respectively, during the whole fetal period studied (Figures 3 and 4).

The linear regression analysis demonstrated a more intense growth rate of nerves in the corpus spongiosum during the 2nd trimester of gestation, when compared with the nerves in the corpora cavernosa. Also, the linear regression analysis demonstrated a more intense growth rate of vessels in the corpus spongiosum when compared with the corpora cavernosa, during the whole fetal period studied.

In addition, the linear regression analysis demonstrated that the nerves grow in a more intense rate than the growth of the area of the penis, both of the corpora cavernosa and corpus spongiosum, during the whole fetal period studied (Figure-5).

**Figure 5A:**
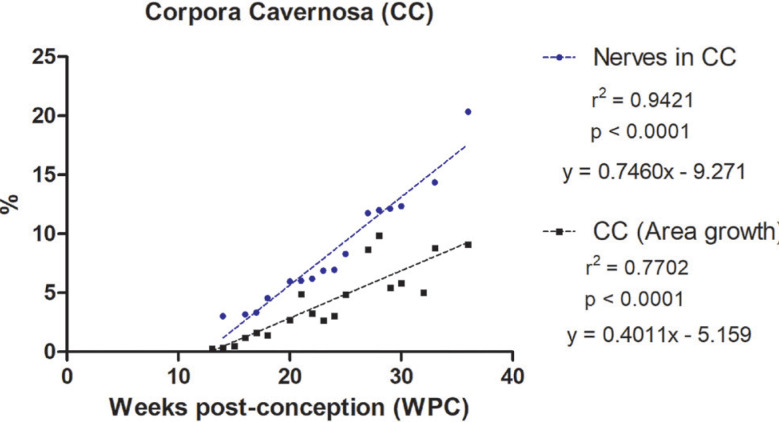
Linear regression analysis showing the correlation of number of Nerves versus Area Growth in the Corpora Cavernosa. Nerves presented a significant and positive correlation with the area of the corpora cavernosa. The rate of nerves growth in the corpora cavernosa was 2.5 times greater, on average, during the entire period studied, when compared to the growth of the area of the corpora cavernosa.

**Figure 5B:**
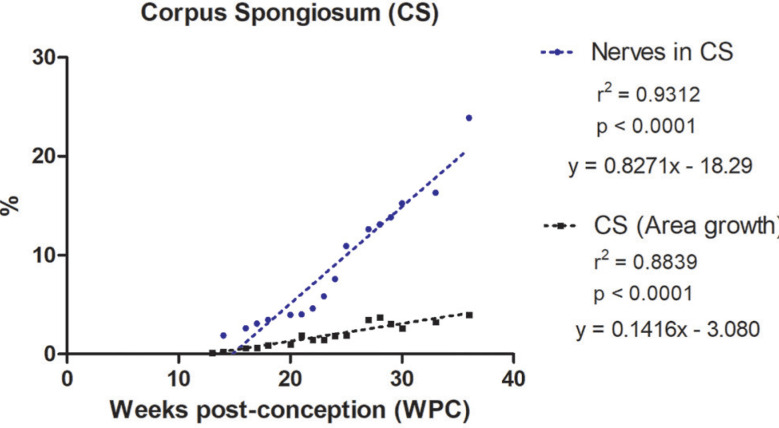
Linear regression analysis showing the correlation of number of Nerves versus Area Growth in the Corpus Spongiosum. Nerves presented a significant and positive correlation with the area of the corpus spongiosum. The rate of nerves growth in the corpus spongiosum was 4.5 times greater, on average, during the entire period studied, when compared to the growth of the area of the corpus spongiosum.

Also, the linear regression analysis demonstrated that vessels grow in a more intense rate than the growth of the area of the penis, both of the corpora cavernosa and corpus spongiosum, during the whole fetal period studied (Figure-6).

**Figure 6a:**
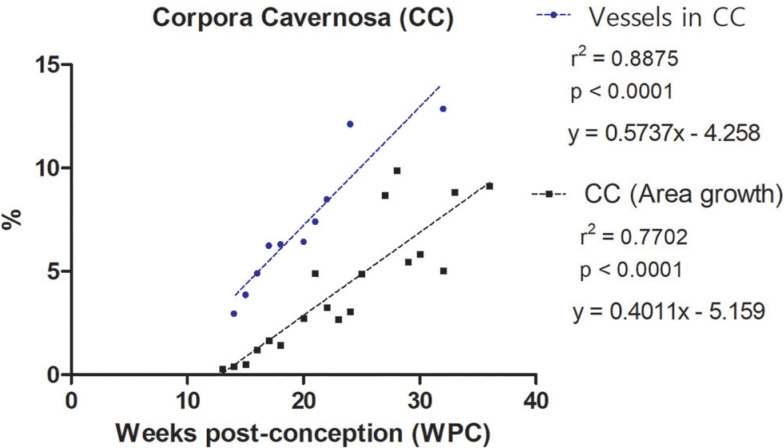
Linear regression analysis showing the correlation of number of Vessels versus Area Growth in the Corpora Cavernosa. Vessels presented a significant and positive correlation with the area of the corpora cavernosa. The rate of vessels growth in the corpora cavernosa was 4.1 times greater, on average, during the entire period studied, when compared to the growth of the area of the corpora cavernosa.

**Figure 6B:**
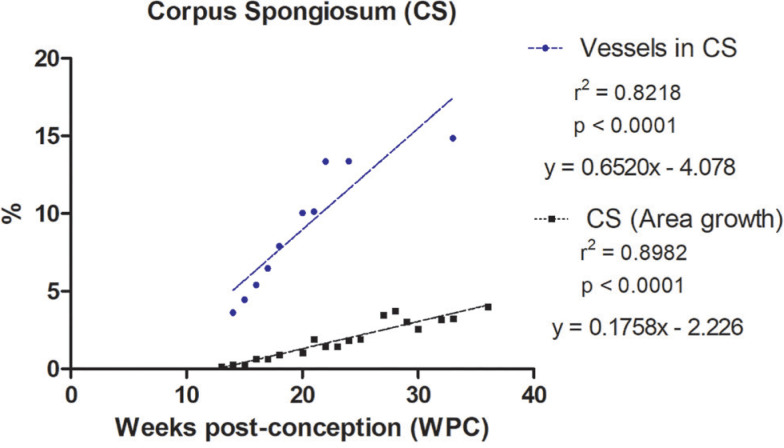
Linear regression analysis showing the correlation of amount of Vessels versus Area Growth in the Corpus Spongiosum. Vessels presented a significant and positive correlation with the area of the corpus spongiosum. The rate of vessels growth in the corpus spongiosum was 9.4 times greater, on average, during the entire period studied, when compared to the growth of the area of the corpus spongiosum.

## DISCUSSION

The identification of alterations in the development of the genitalia during embryonic and fetal period, can lead to an early characterization of other various abnormalities such as genetic diseases and endocrine disorders (20). Furthermore, the surgical correction of penile anomalies is based on knowledge of the anatomy of the penis (21).

Gallo et al. 2014 (11) showed that at 13 weeks post-conception the corpora cavernosa, the corpus spongiosum and the intracavernous septa are already present as well individualized anatomical structures, and, therefore, could be characterized and quantified in the human fetal penis.

The autonomic innervation of the penis derives from the bladder and prostatic plexus, which is composed of the sympathetic nerves L1 and L2, and parasympathetic nerves S2 to S4 (22). In the 13th WPC, the innervation of the corpora cavernosa occupies 14% of the total area, and in the 36th WPC occupies 20% of the total area. In the corpus spongiosum, also in the 13th WPC, the area occupied by the nerves is 8%, and in the 36th WPC is 23%. Therefore, at the end of the third trimester of gestation, the nerves are more numerous in the corpus spongiosum than in the corpora cavernosa. One should take into account that the area of the corpora cavernosa is 9.12mm^2^ in the 36th WPC, while the area of the corpus spongiosum is 3.99mm^2^ (10). This result showed a more intense innervation in the corpus spongiosum than that in the corpora cavernosa at the end of the human gestational period.

Regarding the blood vessels, the absolute area occupied by them is always greater in the corpus spongiosum (3.62% at the 13th WPC and 14.85% at the 36th WPC) than that in the corpora cavernosa (2.2% at the 13th WPC and 12.86% at the 36th WPC) during the whole fetal period.

The results also showed that nerves and vessels, both in the corpora cavernosa and in the corpus spongiosum, have a higher growth rate during the 2nd trimester, when compared with the third trimester. Also, nerves and vessels, grow in a more intense rate than that of the growth of the penile area, during the whole fetal period studied.

The use of ultrasound to determine the patterns of the external genitalia has been used as a tool to determine the sex of the embryo and to characterize the normal patterns of development (23). The different patterns obtained by morphometric analysis of images such as CT, MRI and other methods could be perfectly complemented with structural analysis characterizing microscopically the different structures of the human fetal penis.

Morphological studies showing the embryological development of the tissue components of different organs, and specifically the penis, are few and incomplete. Furthermore, studies using human embryos clearly demonstrated the differences between humans and other animals used as experimental models (24). Rat and mice are often used as laboratory animals for the study of the penis; however, these animals have some disadvantages because the penile structures are different from the human pattern (25). For example, the presence of penile bone, as well as erectile tissue with different structure and distribution. The bone is absent in man and the erectile tissue is predominantly fibrous in rat and mouse, whereas in humans is primarily muscle (25).

This study, therefore, aimed to contribute a line of research that shows the peculiar characteristics of the penis in human fetuses. Also, the study helps to characterize the abnormalities that occur during human development, since it presents a normative pattern of development.

## CONCLUSIONS

In the fetal period, the human penis undergoes major developmental changes, notably in the content and distribution of nerves and vessels. We found strong correlation between nerves and vessels growth with the fetal age, both in the corpora cavernosa and in the corpus spongiosum. There is significant greater proportional number of nerves than vessels during the whole fetal period studied. Nerves and vessels, both in corpora cavernosa and in corpus spongiosum, grow in a more intense rate than that of the growth of the penile area, during the whole fetal period studied.
